# Adversarial robustness in deep neural networks based on variable attributes of the stochastic ensemble model

**DOI:** 10.3389/fnbot.2023.1205370

**Published:** 2023-08-08

**Authors:** Ruoxi Qin, Linyuan Wang, Xuehui Du, Pengfei Xie, Xingyuan Chen, Bin Yan

**Affiliations:** ^1^Henan Key Laboratory of Imaging and Intelligent Processing, PLA Strategy Support Force Information Engineering University, Zhengzhou, Henan, China; ^2^PLA Strategy Support Force Information Engineering University, Zhengzhou, Henan, China

**Keywords:** deep neural network, adversarial robustness, stochastic ensemble, random smoothing, cyberspace security

## Abstract

Deep neural networks (DNNs) have been shown to be susceptible to critical vulnerabilities when attacked by adversarial samples. This has prompted the development of attack and defense strategies similar to those used in cyberspace security. The dependence of such strategies on attack and defense mechanisms makes the associated algorithms on both sides appear as closely processes, with the defense method being particularly passive in these processes. Inspired by the dynamic defense approach proposed in cyberspace to address endless arm races, this article defines ensemble quantity, network structure, and smoothing parameters as variable ensemble attributes and proposes a stochastic ensemble strategy based on heterogeneous and redundant sub-models. The proposed method introduces the diversity and randomness characteristic of deep neural networks to alter the fixed correspondence gradient between input and output. The unpredictability and diversity of the gradients make it more difficult for attackers to directly implement white-box attacks, helping to address the extreme transferability and vulnerability of ensemble models under white-box attacks. Experimental comparison of *ASR-vs.-distortion curves* with different attack scenarios under CIFAR10 preliminarily demonstrates the effectiveness of the proposed method that even the highest-capacity attacker cannot easily outperform the attack success rate associated with the ensemble smoothed model, especially for untargeted attacks.

## 1. Introduction

Deep learning techniques have been successfully applied in various computer vision applications, ranging from object detection (Ren et al., 2016) and image classification (Perez and Wang, 2017) to facial recognition (Parkhi et al., 2015) and autonomous driving (Bojarski et al., 2014) and even in medical computer-aided diagnosis (Hu et al., 2020; You et al., 2022). In these application scenarios, deep learning can be used as an enhancement technique for real data as an artificial intelligence generated content (AIGC) technique to improve performance on the one hand, and as a tool to generate false data to degrade the performance of the model on the other. However, with the increasing use of deep neural networks (DNNs) in various application areas, such as facial recognition technology, for encryption applications, autonomous driving technology for road safety, and computer-aided diagnosis for life safety, there is an urgent need to principally ensure effective defense against security threats, not just the good performance.

The studies on adversarial samples reveal the extreme vulnerability of deep networks, making the study of their robustness even more urgent for security applications. In, Szegedy et al. (2014) discovered that the input-to-output mappings learned by DNNs are generally discontinuous so that even small perturbations in some network inputs can lead to high misclassification errors, which are known as adversarial samples. As a result, many adversarial learning methods similar to cyberspace security games have been developed for both the attack and defense sides. Research on attack and defense in DNN primarily focuses on adversarial samples because of their proactive role in attack and defense games (Akhtar and Mian, 2018; He et al., 2020).

The development of attack methods is constantly intertwined with the proposal of defense methods. Both types of methods act as opposing sides in a competitive game, developed in a mutually promoting and closely reciprocal process. Certified defense methods are supported by rigorous theoretical security guarantees that obtain a robustness radius under the Lp distortion constraint (Fischetti and Jo, 2017). Nevertheless, these certified defense methods are still not widely used in DNN architectures on big data through exact or conservative approaches. More flexible and effective defense methods are empirical methods based on assumptions and experimental results (Papernot et al., 2016; Lakshminarayanan et al., 2017; Kurakin et al., 2018). Although empirical defense methods are convenient, they have practical limitations in their applicability, which may result in attackers generating more challenging adversarial samples to break the defense.

The rapid development of attack algorithms and extensive research on empirical defenses eventually led to the game of attack and defensive in deep learning files. For example, the distillation method (Papernot et al., 2016) which uses gradient shielding to prevent white-box attacks, is not effective against the CW attack (Carlini and Wagner, 2017). The model ensemble method (Lakshminarayanan et al., 2017) was initially proposed as a defense method but has been found to be ineffective (He et al., 2017) and is now commonly used as an attack method to improve the transferability of adversarial samples (Tramèr et al., 2018). The nature and wide applicability of empirical defense methods have sparked intense competition with attack methods. However, defense methods are primarily passive.

**Figure 1:**
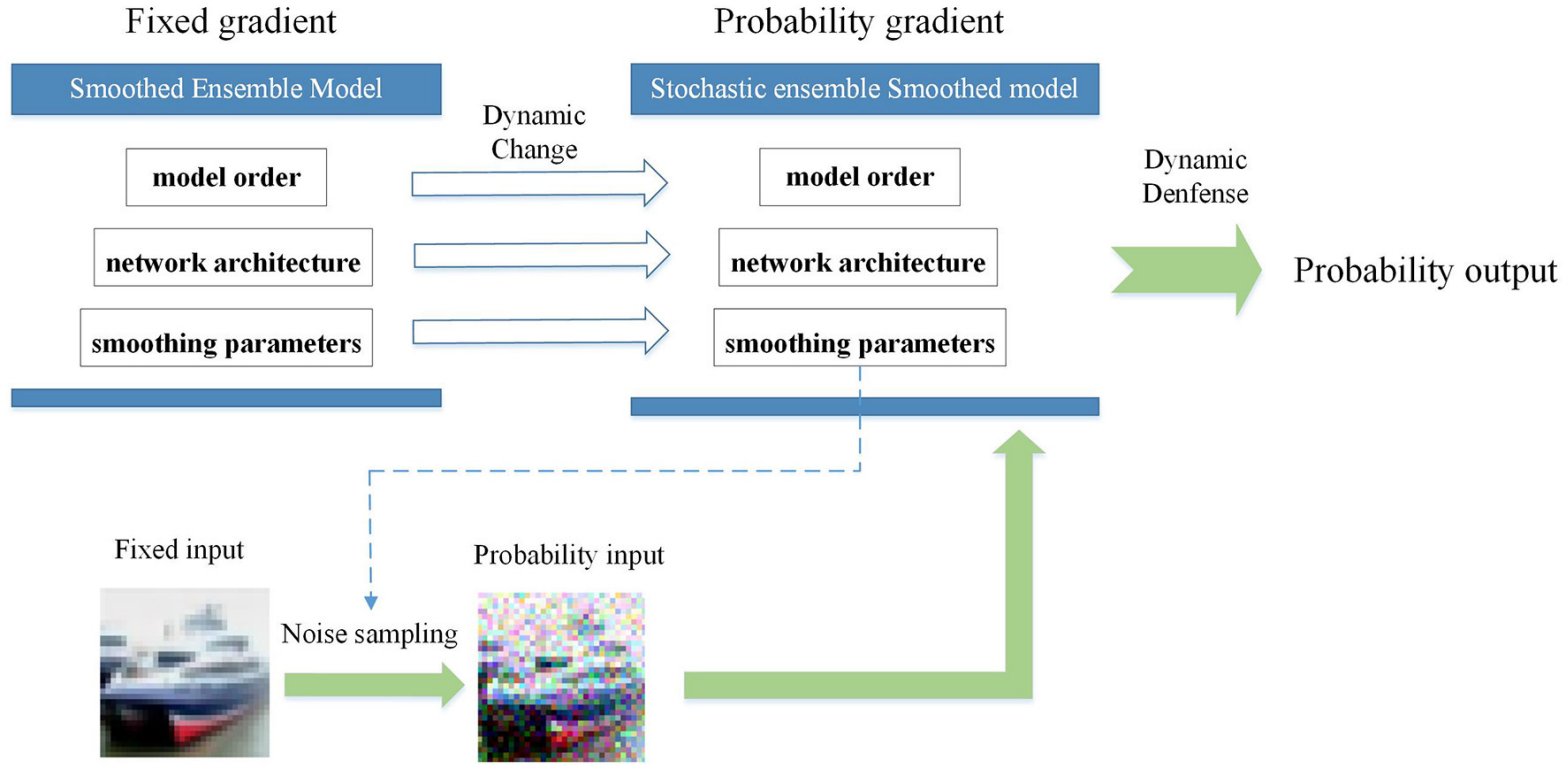
A graphical illustration of the proposed variable attributes of ensemble strategy for adversarial robustness of deep neural networks.

According to theoretical developments in cybersecurity, the two sides in a competitive game without a strongly secure defense method will eventually reach a Nash equilibrium (Attiah et al., 2018). To address this challenge, generalized robust-control defense methods, such as moving target defense (MTD) (Jajodia et al., 2011) and dynamic defense model (DDM) (Wu et al., 2019; Wu, 2020), have been proposed with probabilistic formulations of the network attributes. The inherent randomness and unpredictability of the system make it more difficult for the attacker to detect, highlighting the importance of the same defense approach applied in DNNs. Recent research on adversarial robustness indicates that adversarial examples are inevitable for DNNs. This article starts from the premise of learning from the development experience of cybersecurity under the current technical levels and treating the classification problem based on deep neural networks as a whole system rather than a single model. In the case where effective adversarial samples mostly depend on specific model information while the adversarial transferability needs to be improved, this article proposes an attribute-based stochastic ensemble model using the DDM ideology to combine randomness with model diversity. In the proposed method, the ensemble quantity, network architecture, and smoothing parameters are used as ensemble attributes to dynamically change it before each inference prediction request. As shown in Figure 1, these variable attributes of the ensemble model represent a more active and generalized defense approach to overcome the limitations of empirical and deterministic defense at the current stage. In summary, the main contributions of our study are as follows:

1. Facing the endless arms race of adversarial attack and defense, this article proposes an attribute-based stochastic ensemble model using the DDM ideology to combine randomness with model diversity. A more diverse collection of heterogeneous and redundant models is created for the ensemble, accounting for variations in ensemble attributes and dynamically changing structures for each inference prediction request at the model level, hoping to further change the passive position of the defense at this stage.
2. For the robustness evaluation of the proposed method, this article considers the attack and defense game idea as a starting point, assuming that the attacker knows the defense strategy, and simulates a series of possible adversarial game processes for a more comprehensive evaluation. The different capabilities of the attack scenario are set up and the potential defense risks are assessed using attack success rate versus distortion (*ASR-vs.-distortion curves*) based on Monte Carlo simulations.
3. We analyze different robustness results under attack scenarios and algorithms with various capabilities and identify important conditions for the proposed method to exert its advantages in practice. The experimental results under CIFAR10 show that even the most capable attacker is unable to outperform the best result under current random-based methods, demonstrating the effectiveness of the proposed method in attack and defense games.

## 2. Related work

### 2.1. Defense method based on input randomization

Recently, theoretical guarantees for the robustness of DNNs have been gradually combined with relevant aspects of cybersecurity. Random smoothing was originally proposed based on the intention of differential privacy (Lecuyer et al., 2019) from cyberspace defense methods to prevent the attackers from obtaining exact gradient information by adding random noise to the input image during training and testing (Cohen et al., 2019; Lecuyer et al., 2019; Li B. et al., 2019). Random self-ensemble (RSE) (Liu et al., 2018) and Smoothed WEighted ENsemble (SWEEN) (Liu et al., 2020) improve the adversarial robustness by combining the randomness properties in the case of the ensemble. Unlike these previous studies, this study is inspired by the DDM ideology in cyberspace security and sets the model parameters on which the attack conditions directly depend as the objects of randomization to further improve the adversarial robustness under ensemble conditions.

### 2.2. Defense methods based on diversified ensemble networks

In addition to the gradient shielding effect of the random smoothing, the robustness provided by ensemble models also depends on the diversity of the sub-model (Lakshminarayanan et al., 2017). Constraints on the gradient diversity of sub-models mostly depend on empirical conclusions about the diversity of model architecture (Kurakin et al., 2018) or the training hyperparameters (Wenzel et al., 2020) and gradient diversity between sub-models (Pang et al., 2019). Unlike the fixed ensemble of diverse sub-models in these methods, this study uses the empirical conclusions of model attributes to contrast the diverse sub-models. By randomly selecting these attributes, this method combines diversity and randomization characteristics to improve adversarial robustness under the ensemble condition.

### 2.3. Adversarial samples and robustness evaluation

Attack algorithms can be divided into white-box and black-box methods based on their capabilities (Akhtar and Mian, 2018). White-box methods rely on full knowledge of the network gradients. The fast gradient sign method (FGSM) (Goodfellow et al., 2015) is a basic and effective method that generates adversarial samples by adding the sign reverse of the gradient to the original images. Based on attack performance and transferability, iteration-based approaches include the basic iterative method (BIM) (Kurakin et al., 2016), momentum iterative method (MIM) (Dong et al., 2018), and projected gradient descent method (PGD) (Madry et al., 2018). In contrast, black-box attackers have no knowledge of the network gradients that can be divided into query-based and transfer-based methods. The query-based method achieves gradient estimation by querying the output of the target model including natural evolution strategies (NES) (Ilyas et al., 2018), simultaneous perturbation stochastic approximation (SPSA) (Uesato et al., 2018), and NATTACK (Li Y. et al., 2019). The transfer-based method generates adversarial samples by constructing substitution models, usually using the ensemble model constructed by normally trained sub-models (Tramèr et al., 2018) or shadow model (Zhang et al., 2022). In previous studies, different adversarial sample generation algorithms can verify the different performances of the defense method from different perspectives. Unlike the previous single analysis of the defense capability under optimal attack algorithms, this study considers the game-like nature of the attackers and designs more diverse attack and defense scenarios under random conditions to fully verify the effectiveness of the proposed method.

## 3. Materials and methods

This study focuses on the image classification task of CIFAR10 (Krizhevsky and Hinton, 2009) for preliminary verification. Section 3.1 first introduces the basic method of random smoothing and shows the relationship with the proposed stochastic ensemble model to theoretically demonstrate that the proposed method achieves a certified robust radius no less than the state-of-the-art (Liu et al., 2020) under the random conditions. Furthermore, the empirical diversity requirement between sub-models in the ensemble is characterized by attribute-based heterogeneous redundant models to improve the robustness of the stochastic ensemble model in Section 3.2. Finally, Section 3.3 outlines the strategy for a stochastic ensemble approach with variable attributes.

### 3.1. Preliminaries of stochastic ensemble modeling

Let the random smoothing model *g* be trained by a basic classifier *f* by sampling, adding the noise δ~*N*(0, σ^2^*I*) to the input images and minimizing the corresponding classification losses (Cohen et al., 2019; Lecuyer et al., 2019; Li B. et al., 2019). For the model prediction in the training and testing process, the output of random smoothing model *g* is defined as a mathematical equation as follows:


(1) $$\begin{array}{l} g\left(x\right)=E_{\delta \sim N\left(0,\sigma^{2}I\right)}\left[f\left(x+\delta \right)\right] \end{array}$$

An ensemble model *f*_*ens*_ containing K models obtains the final prediction by summing the function outputs of the individual candidate models. The mathematical representation of the ensemble model can be written as follows:


(2) $$\begin{array}{l} f_{ens}\left(x,\theta \right)=\overset{K}{\underset{k=1}{\sum }}f\left(x,\theta_{k}\right) \end{array}$$

The SWEEN approach creates an ensemble-smoothed model with a weight parameter ω for each model, which improves the provable robustness radius (Liu et al., 2020). In terms of the probability distribution of the input noise, the predicted output of the SWEEN model is given by a mathematical expectation operator as follows:


(3) $$\begin{array}{l} SWEEN=E_{\delta }\left[\sum_{k=1}^{K}\omega_{k}f\left(x+\delta ;\theta_{k}\right)\right]=\sum_{k=1}^{K}\omega_{k}E_{\delta }\left[f\left(x+\delta ;\theta_{k}\right)\right]\\ \;=\sum_{k=1}^{K}\omega_{k}g\left(x;\theta_{k}\right) \end{array}$$

The constant weight parameters ω of the candidate models are independent of the SWEEN model output and can be optimized as ω^*^. Unlike SWEEN, the ensemble attributes of the proposed stochastic ensemble model (SEM) are randomly adjusted to dynamically structure the ensemble model at each time inference prediction request making the output of candidate models in SEM have an additional mathematical expectation in terms of probability of occurrence. However, the probability of occurrence of a particular candidate model under the SEM is assumed to be determined by the expectation *E*(_*f*_*k*_)*occurrence*_ = ω_*k*_ and statistically independent of the prediction expectation. Therefore, as shown in Equation (4), the stochastic ensemble and SWEEN models can be equivalent in terms of output expectations. The theoretical improvement of the robustness radius by the SWEEN model (Liu et al., 2020) is a special case of the SEM. By controlling the probability of the occurrence of sub-models, the SEM can theoretically achieve well-certified robustness. However, more importantly, such changes based on the model level improve the dynamic properties of the ensemble and achieve a more generalized dynamic change of the model gradient in each inference prediction:


(4) $$\begin{array}{l} \text{SEM}=E[\sum_{k=1}^{K}f_{k}(x+\delta ;\theta_{k})]=\sum_{k=1}^{K}E{\left(f_{k}\right)}_{apparence}\\ \;\;\;\;\;\;\;\;\;\;\;\times E[f_{k}(x+\delta ;\theta_{k})]\\ \text{SEM}=\sum_{k=1}^{K}\omega_{k}^{*}E[f_{k}(x+\delta ;\theta_{k})]=\sum_{k=1}^{K}\omega_{k}E[f_{k}(x+\delta ;\theta_{k})]\\ =\text{SWEEN}\;\text{when}\;\omega_{k}=\omega_{k}^{*} \end{array}$$

### 3.2. Attributes-based heterogeneous redundant models

The application of random input to the sub-model parameters in SWEEN (Liu et al., 2020) improves the certified robustness of the ensemble. The analysis in Section 3.1 has shown that these sub-models can also serve as a random condition, expanding randomness at the model level without compromising the certified robustness. According to previous empirical defense conclusions, the diversity between sub-models enhances the robustness of the ensemble condition (Pang et al., 2019; Wenzel et al., 2020). Moreover, diversity is also the DDM property in cybersecurity (Wu et al., 2019). Therefore, the first step for the proposed variable attribute-based SEM is a collection of heterogeneous redundant sub-models. In addition to the diversity of the model architectures (Kurakin et al., 2018), different hyperparameters for optimizing the sub-models can also have different effects on the convergence of the gradient (Wenzel et al., 2020). Random smoothing hyperparameters for a variety of noise parameters in training further enhance model redundancy and diversity within the same architecture. The proposed SEM uses network architecture, depth, and width as well as smoothing parameters as variable ensemble attributes. In Section 4.5, we present detailed experimental results on the influence of model architecture and other parameters.

The heterogeneous redundant model collection is obtained by separately training a smoothed model on the CIFAR10 dataset (Krizhevsky and Hinton, 2009; Hendrycks et al., 2019). The variable ensemble attributes in this study include architectures of different depths and widths. Table 1 shows the *approximated certified accuracy* (ACA) of the predictive performance of each sub-model. The models marked in red did not meet performance requirements and were excluded from subsequent experiments. Although some simple models, such as AlexNet and shallow VGG, were unable to achieve stable smoothed prediction, unsmoothed models were used for the SEM. The experimental results in Section 4.5 further demonstrate that the heterogeneity of the model collection plays a crucial role in the robustness of the stochastic ensemble.

**Table 1:** Heterogeneous redundant model collection on CIFAR10.

**Model architecture**	**Smoothing parameter** σ			**Model architecture**	**Smoothing parameter** σ		
	**0.25**	**0.75**	**1.5**		**0.25**	**0.75**	**1.5**
**DenseNet** (Gao et al., 2017)				**VGG** (Simonyan and Zisserman, 2014)			
DenseNet100 (95.5)	94.03	89.96	83.56	VGG11 (92.1)	9.99	80.11	20.88
DenseNet121 (94.1)	91.23	87.01	82.08	VGG13 (94.3)	65.67	10.0	61.18
DenseNet161 (94.2)	92.31	87.88	82.80	VGG16 (93.9)	9.99	9.99	9.99
DenseNet169 (94.0)	91.29	87.96	81.11	VGG19 (93.3)	91.83	87.50	81.74
**WRN** (Zagoruyko and Komodakis, 2016a) (96.2)	91.78	90.23	83.43	**AlexNet** (Krizhevsky et al., 2017) (77.2)	9.99	9.99	9.99
**ResNet** (He et al., 2016)				**InceptionV3** (Szegedy et al., 2016) (93.8)	91.91	86.86	80.38
ResNet18 (93.3)	90.49	86.63	80.15	**MobileNetV2** (Sandler et al., 2018) (94.2)	88.91	84.74	77.35
ResNet34 (92.9)	91.20	87.20	81.76	**ResNext** (Xie et al., 2017) (96.2)	93.12	88.70	80.62
ResNet50 (93.9)	91.16	86.29	80.28	**GoogleNet** (Szegedy et al., 2015) (92.7)	91.63	87.61	80.64

### 3.3. Stochastic ensemble with variable attributes

In a model ensemble, temporal gradient variations result from attribute-based gradient changes in each smoothed model. This article proposes a stochastic ensemble strategy based on heterogeneous redundant models, where each prediction is made by the stochastic selection of ensemble attributes. The randomness of the model attributes reflects SEM randomness, which varies in the frequency of the ensemble quantity, network architecture, and smoothing parameters when multiple requests for gradient or output information are made. The model randomly selects the number of sub-models for the ensemble. Once the number of ensemble models has been determined, the model stochastically selects the model architecture from Table 1. Next, it randomly selects various parameters of the selected model architecture, such as network depth and smoothing parameters. Finally, the ensemble model is determined based on these stochastic ensemble attributes. Algorithm 1 provides a detailed explanation of the selection process for this method.

**Algorithm 1:** Framework of the stochastic ensemble for the defense system.

**Require**: Image x for classification, K-ensemble quantity, f-model architecture, δ-smoothing parameter; *f*_*k*_(*x*+δ)-model source output before softmax
**Ensure:** *output*_*ensemble*_-softmax operation of ensemble model
1. **While** inference prediction request for one user **do**
2. Randomly determine the model quantity K for the ensemble;
3. Randomly select the number of model architectures *f* according to model quantity K;
4. Randomly select different smoothing parameters δ for each model architecture, the sub-model of ensemble is determined by *f*_*k*_ finally;
5. *source*_*ensemble*_←0
6. **for** each *k*∈[1, *K*]**do**
7. *source*_*model*_←*f*_*k*_(*x*+δ)
8. *source*_*ensemble*_←*source*_*ensemble*_+*source*_*model*_
9. **end for**
10. *output*_*ensemble*_←*softmax*(*source*_*ensemble*_)
11. **end while**

**Figure 2:**
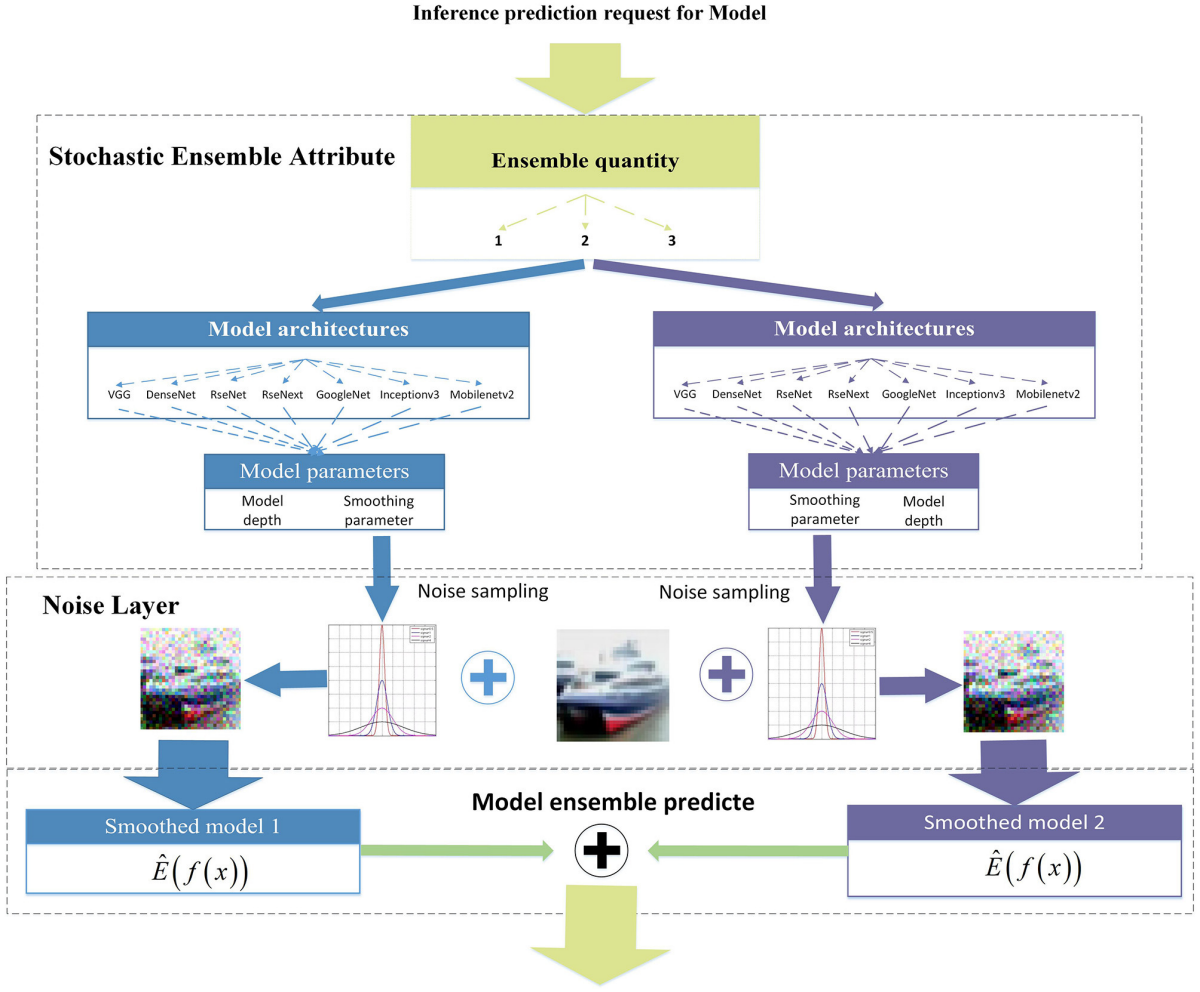
A flowchart of the stochastic ensemble smoothing strategy.

Figure 2 shows a flowchart of the stochastic ensemble strategy. By incorporating the model architecture into ensemble attributes, each iteration of the ensemble incorporates gradient differences based on changes in the network architecture. In addition, network depth and smoothing parameters were used as ensemble attributes to increase ensemble diversity. The number of sub-models in each ensemble iteration is relatively small [set as (1–4) in this article] compared to all of the model collections to ensure gradient differentiation. On the one hand, a larger number of sub-models sets in each ensemble iteration will reduce the ensemble diversity and gradient variations. On the other hand, a large number of sub-models sets in the ensemble will lead to improved transferability of adversarial samples generated from a possible white-box attack for a single ensemble iteration. For probabilistic ensembles, allowing a single model in the stochastic state does not affect the mathematical expectation of the prediction, but ensures a diversity gradient change in each ensemble iteration. The attribute of the ensemble quantity plays a key role and has an important impact on robustness, which will be discussed in detail in Section 4.5.

The SEM introduces the dynamic nature of DNNs through the stochastic selection of the ensemble attributes. The dynamic changes reflect the random distribution of input noise and probabilistic gradient information during each ensemble iteration. Essentially, the randomness of ensemble attributes shields the gradient information and increases the confusion under white-box and query-based black-box attacks.

## 4. Experiments and results

Currently, most single static models rarely consider both white-box and black-box attack robustness evaluation comprehensively but consider white-box attack robustness as the evaluation metric. The probabilistic gradient of the proposed SEM makes it difficult for attackers to fully discover the model parameter of each particular ensemble iteration. From the attackers' point of view, the more effective attack is no longer the white-box attack defined in the original evaluation but is based on the attacker's knowledge of the model collection to achieve the black-box attack or approximate white-box attack. This section comprehensively designs different knowledge of attacker against the SEM and comprehensively illustrate the potential and drawbacks of the proposed method. To define and evaluate the robustness under random conditions, the attack success rate is further defined as a potential risk by *ASR-vs.-distortion* curves (Dong et al., 2019) based on Monte Carlo simulations. For the conclusion of robustness, this section generally verified and evaluated adversarial robustness same as the definition in cyberspace security: the most capable attacker for SEM cannot easily outperform the best result under current random-based methods.

### 4.1. Attack success evaluation metrics based on empirical risk

The *ASR-vs.-distortion* curves are generated by an optimal search of the adversarial perturbation budget (Dong et al., 2019). Due to the random condition, the Monte Carlo simulation is used for approximate evaluation as in random smoothing (Cohen et al., 2019). Each adversarial sample *x*_*adv*_ is hard-predicted N times by the SEM, and the most predicted category is considered the output category with the highest probability. The baseline accuracy of the clean sample through this simulation is 93.4**%**. Compared with the according accuracy result of the single smoothing model in Table 1, there is no damage but even improvement for clean-sample prediction. The attack success rate with the adversarial sample x is given as follows:


(5) $$\begin{array}{l} Succ\left(C,A_{\varepsilon ,p}\right)\\ =\left\{ \begin{array}{l} \frac{1}{N}{\left(\sum_{n=1}^{N}{\left(\sum_{k=1}^{K}g_{k}\left(A_{\varepsilon ,p}\left(x\right)\right)\right)}_{one\_hot}\right)}_{\max}\neq y\text{ untargeted}\\ \frac{1}{N}{\left(\sum_{n=1}^{N}\left({\sum_{k=1}^{K}g_{k}\left(A_{\varepsilon ,p}\left(x\right)\right)}_{one\_hot}\right)\right)}_{\max}=y_{t}\text{ targeted} \end{array}\right. \end{array}$$

The attack success probability is redefined as the proportion of Monte Carlo simulations in which each *k*-th iteration model *g*_*k*_ outputs the target category for the given adversarial sample *A*_ε, *p*_ with a perturbation budget ε under the *l*_*p*_ norm. This probability is estimated using class count statistics obtained by one-hot encoding of the category probability vector, and then converting each predicted value to its equivalent probability using a probability conversion function. Such probabilities can be used in a two-sided hypothesis test that the attack success rate conforms to the binomial distribution *n*_*succ*_~*Binomial*(*n*_*succ*_+*n*_*nonsucc*_, ρ) as follows:


(6) $$\begin{array}{l} Succ\left(C,A_{\varepsilon ,p}\right)\\ =\left\{ \begin{array}{l} \frac{1}{N}{\left(\sum_{n=1}^{N}{\left(\sum_{k=1}^{K}g_{k}\left(A_{\varepsilon ,p}\left(x\right)\right)\right)}_{one\_hot}\right)}_{\max}\neq y\text{ or }\\ \frac{1}{N}{\left(\sum_{n=1}^{N}{\left(\sum_{k=1}^{K}g_{k}\left(A_{\varepsilon ,p}\left(x\right)\right)\right)}_{one\_hot}\right)}_{\underset{c\neq y}{\max}\;}\geq \alpha \text{ untargeted}\\ \frac{1}{N}{\left(\sum_{n=1}^{N}{\left(\sum_{k=1}^{K}g_{k}\left(A_{\varepsilon ,p}\left(x\right)\right)\right)}_{one\_hot}\right)}_{\max}=y_{t}\text{ or }\\ \frac{1}{N}{\left(\sum_{n=1}^{N}{\left(\sum_{k=1}^{K}g_{k}\left(A_{\varepsilon ,p}\left(x\right)\right)\right)}_{one\_hot}\right)}_{t}\geq \alpha \text{ targeted} \end{array}\right. \end{array}$$

The abstention threshold α is a parameter used to limit the probability of returning an incorrect prediction in order to control potential empirical model risk (Hung and Fithian, 2016). A value of α directly affects the *ASR-vs.-distortion* curves. In this case, the threshold α is set at 0.3 to evaluate the random smoothing model.

### 4.2. Attack scenarios

In this section, the attacker's knowledge of the SEM attributes is discussed in detail and the attack scenarios are designed to fully characterize the robustness of the proposed method. By comparing the robustness evaluation results of attackers with different capabilities under the proposed method with the results of the contrast models, the attack scenarios are designed to discuss two aspects of robustness: first, under which attack capabilities is the proposed method most vulnerable and which is the most robust. This will help defenders to understand which attributes are important for protection. Second, whether the proposed method is robust enough such that even an attacker with the highest attack capability cannot easily exceed the attack success rate associated with the best contrast method (Athalye et al., 2018).

In the random condition, different attackers can have different degrees of knowledge about the model collection, but no knowledge about the current ensemble state. From an attack point of view, the attacker should use a white-box attack under expectation, a transfer-based attack under the substitution model, or a query-based black-box attack. The attacker's capabilities are determined by the knowledge of the model collection and the ensemble attributes, as outlined from high to low in Table 2. In the white-box attack under expectation, attackers A and B have full knowledge of model collection and are implemented as Expectation Over Transformation (EOT) attack method (He et al., 2017; Croce et al., 2022) white-box attack according to the different expectation estimation iteration. In the transfer-based attack under the substitution model, attackers C and D have partial knowledge of the model collection and are defined according to the different transfer strategies. In addition, attacker E uses the query-based black-box attack algorithm. The analysis of our experimental setup highlights the varying ability of the A–D attackers to approximate the gradient distribution expectation, which comprehensively illustrates the robustness of our method under more complicated conditions.

**Table 2:** The definition of the attacker's ability from high to low and the contrast method.

**Attacker tag**		**Definition**	**Contrast method**		**Definition**
White-box attack as EOT	Attacker A	The attacker has full knowledge of the model collection and can obtain ensemble attributes in real-time. However, they lack the ability to predict these attributes for the next ensemble iteration, where their best strategy is to implement the EOT attack on each ensemble iteration for the expectation of gradient.	Under White-box attack	Contrast method F	The ensemble smoothed model under a white-box attack
				Contrast method G	The single-smoothed model under a white-box attack
	Attacker B	The attacker has full knowledge of the model collection but cannot obtain ensemble attributes in real-time, where their one of the attack strategies is to implement an EOT attack on periodic ensemble iteration.		Contrast method H	The ensemble model under a white-box attack
				Contrast method I	The single model under a white-box attack
Transfer-based black-box attack	Attacker C	The attacker has knowledge of half of the models in the collection for the experiment. Their best attack strategy is to structure the alternative SEM model on known models as an EOT method for generalized adversarial samples.	Under Black-box attack	Contrast method J	The ensemble smoothed model under the black-box attack
				Contrast method K	The smoothed model under the black-box attack
	Attacker D	The attacker has knowledge of half of the models in the collection. Their more direct attack strategy is to use all the known models as an ensemble model to generate transfer adversarial samples.		Contrast method L	The ensemble model under the black-box attack
Query-based black-box attack	Attacker E	The attacker lacks any knowledge of the model collection or gradients and can only query the model probability vector to implement a black-box attack.		Contrast method M	The single model under the black-box attack

### 4.3. Experimental settings of competitive baseline methods

**Figure 3:**
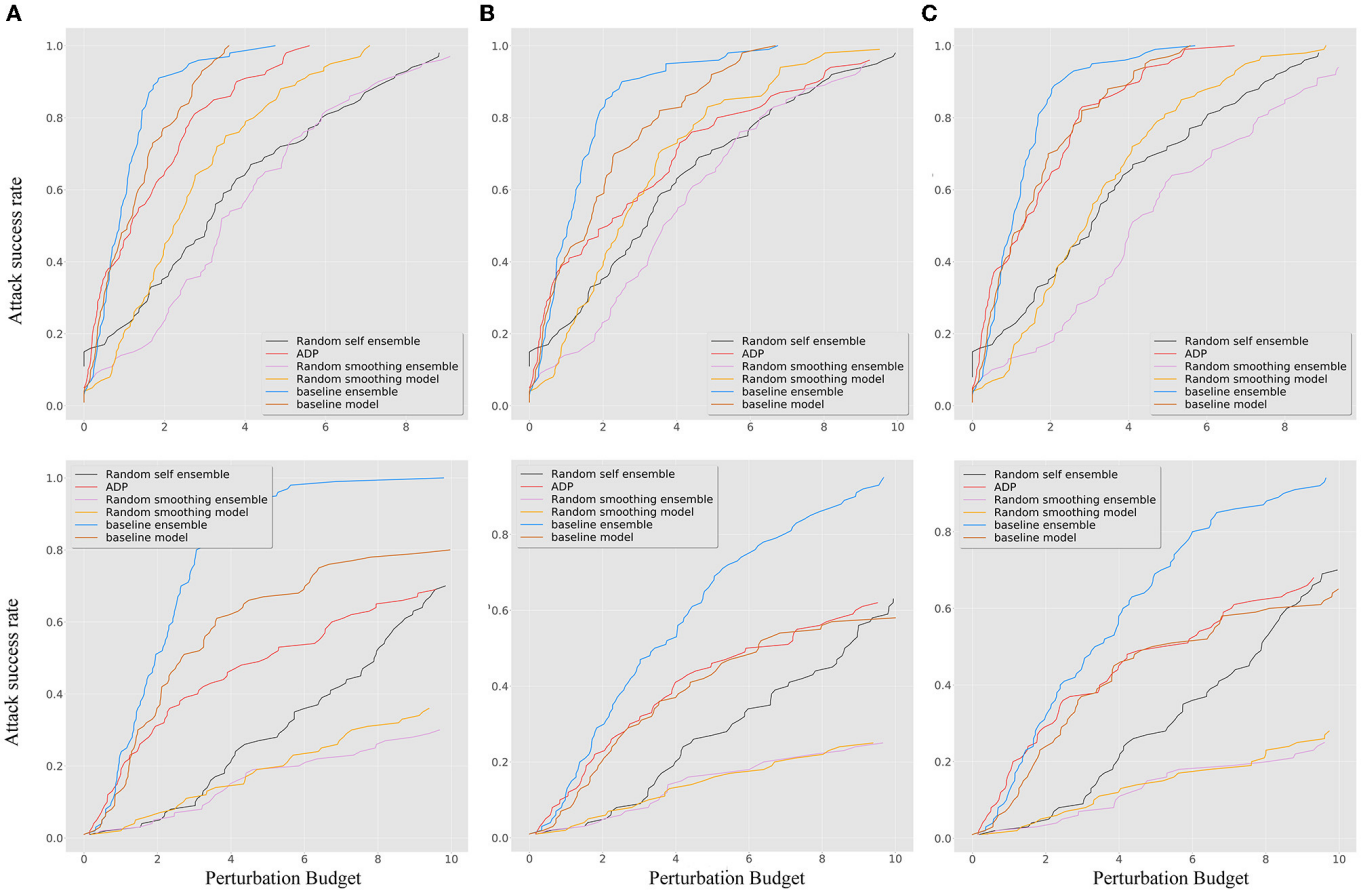
The *ASR-vs.-distortion* curves for different ensemble baseline methods under untargeted (first line) and targeted (second line) white-box attacks: **(A)** BIM; **(B)** MIM; and **(C)** PGD.

To verify the improvement of robustness, several ensemble methods were selected as baselines for comparison, including RSE (Liu et al., 2018), random smoothing (Liu et al., 2020), and the adaptive diversity promoting (ADP) (Pang et al., 2019). For the details of the experiment, both the random smoothing ensemble and baseline ensemble method used three different model architectures, namely, DenseNet100, ResNet50, and WRN, as shown in Table 1, which perform better on clean datasets. The parameters of the smoothed models were chosen as Gaussian noise with δ 0.25. Figure 3 shows that neither the ADP nor the RSE methods outperform the ensemble-smoothed method. Among the defenses based on randomness and ensemble diversity, the ensemble smoothed model has SOTA results at this stage and structure as the contrast method F in attack scenarios. In a follow-up experiment, the random smoothing-related method with the best robustness is used as a contrast method (corresponding to the four curves of F, G, J, and K in the contrast methods as shown in Table 2) to demonstrate the performance of the proposed method for brief.

### 4.4. Robustness analysis based on the attack scenario

A comprehensive evaluation of adversarial robustness can be achieved by considering different combinations of attack capabilities, methods, targets, and perturbation constraints. Further attacks are carried out by the algorithm using three standard methods (BIM, MIM, and PGD) with attackers *A, B, C*, and *D* and contrast methods *F, G, H*, and *I*, respectively. In addition, NES and SPSA attacks were used in conjunction with contrast methods E, G, K, L, and M. For all *ASR-vs.-distortion* curves, the search step was set to 10 while the binary search step was set to 20. For the white-box attacks, the number of attack iterations of both the BIM and MIM was set to 20, while for the query-based black-box attacks, the maximum number of queries was set to 5000. The following experiments aim to evaluate the proposed methods and analyze the defense characteristics of dynamics under different attack scenarios set in Section 4.2.

#### 4.4.1. Transfer-based and white-box attack analysis

**Figure 4:**
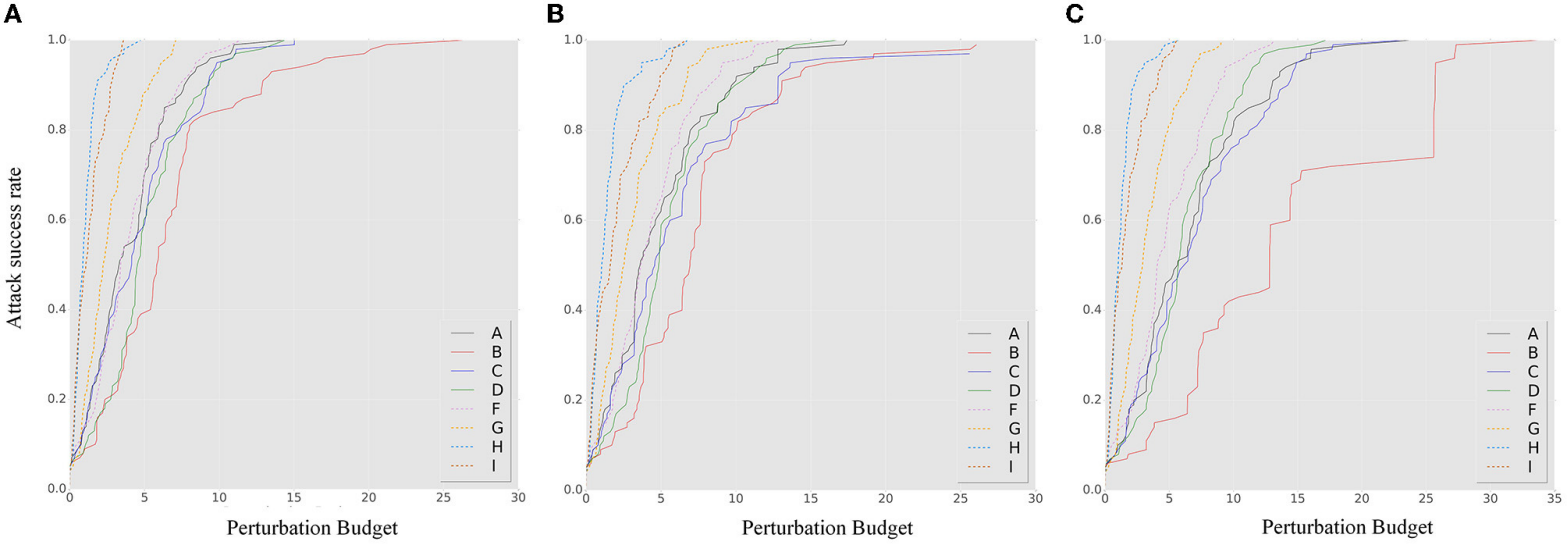
The *ASR-vs.-distortion* curves for untargeted transfer-based white-box attacks: **(A)** BIM, **(B)** MIM, **(C)** PGD. The A–D solid lines show the *ASR-vs.-distortion* curves under different attack capabilities while the dashed lines F–I show the curves under the contrast method. Compared to the two curves, the stochastic ensemble has better robustness even under the strongest adversary.

Figure 4 shows the *ASR-vs.-distortion* curves for untargeted transfer-based attacks. *A, B, C*, and *D* represent different attack scenarios, while the contrast methods *F, G, H*, and *I* are shown as dashed curves. Compared to the baseline models, we can observe that the ensemble model is highly vulnerable to white-box attacks, even worse than the single models. The random smoothing method improves the robustness of a single model, and the ensemble-smoothed model further improves the robustness and addresses the vulnerability of the ensemble under white-box attacks. Among all attack methods, attacker *B* has the worst attack performance, indicating that protecting the model from frequent access to gradient information at each iteration is crucial for SEM robustness. Attacker D, who has partial knowledge of the model collection but ensemble attributes in each iteration, can achieve transfer attacks through the ensemble and achieves similar robustness performance (even better than PGD) compared to attacker A. However, comparing the performance of attackers C and D, the SEM does not improve the attack transferability effect as a regularization method. This reveals the importance of protecting the model collection for SEM robustness. When the attacker has a higher transferability attack algorithm (for the MIM and PGD), the benefits of transferability are only for attacker *D* and are no longer attained by SEM. For the ensemble smoothed model (F curves) that has the SOTA performance between the contrasting baseline methods, the best attack performance cannot easily exceed the attack success rate associated with it.

**Figure 5:**
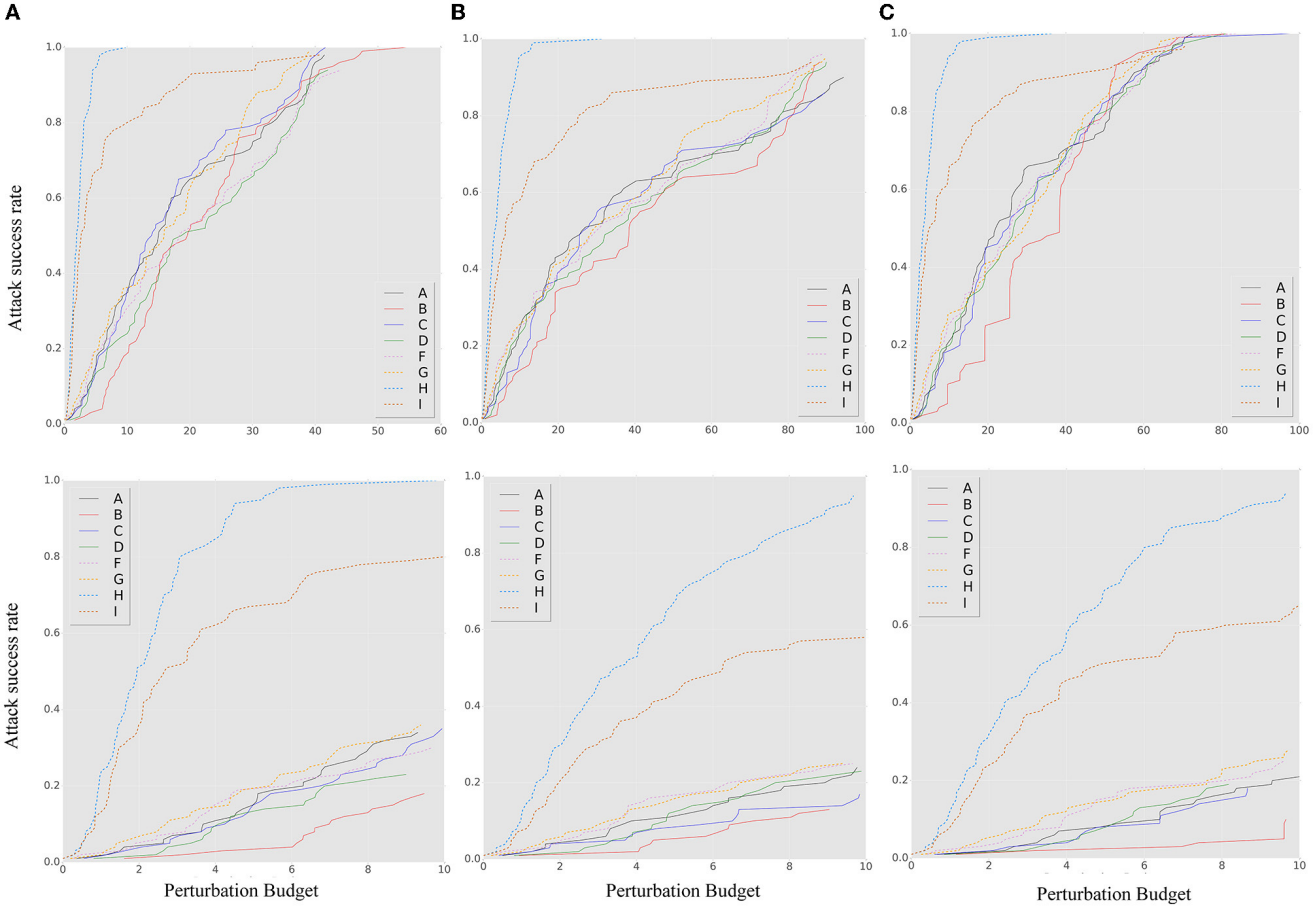
The *ASR-vs.-distortion* curves for targeted transfer-based white-box attacks: **(A)** BIM, **(B)** MIM, and **(C)** PGD. The solid lines A–D show the result under different attack capabilities while the dashed lines F–I show the curves under the contrast method. The second line shows the corresponding detail result with a small perturbation for clarity. From the detailed result, it can be concluded that the SEM exhibits superior robustness performance under conditions of small perturbation.

Figure 5 shows the *ASR-vs.-distortion* curves for targeted transfer-based white-box attacks. When comparing different attack algorithms, the improved transferability of the PGD method does not significantly improve the attack performance under SEM. However, its robustness is significantly improved against the momentum-based attack, indicating that the randomness of the gradient at the model level has some impact on the confusion of the gradient direction. The variation in the attack knowledge of model collection between *A* and *C* does not significantly affect the robustness of SEM when against targeted attacks. However, contrary to the conclusion drawn from untargeted attacks, the robustness performance of SEM under *A* and *C* does not consistently exceed that of the ensemble smoothed or single smoothed model, demonstrating the lack of heterogeneity of the model in the gradient direction. However, as the detailed results in the second line of Figure 5 shown, the proposed method consistently demonstrates superior robustness under small perturbations. When comparing attackers *A, B, C*, and *D*, the weakest attack performance is exhibited by B (although this could be reversed when attacker D uses the PGD algorithm). Combined with the results of the untargeted attacks, we suggest that reducing the frequency of ensemble changes is critical for SEM when the model collection and ensemble attributes can be obtained by an attacker.

#### 4.4.2. Query-based black-box analysis

**Figure 6:**
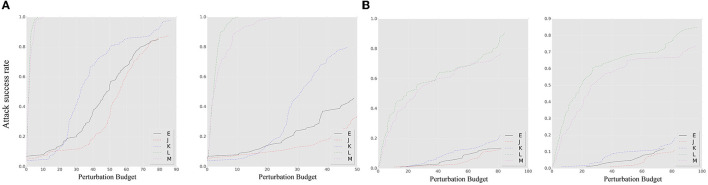
The *ASR-vs.-distortion* curves for source-based black-box attacks: **(A)** untargeted attack; **(B)** targeted attack. The left side of each attack target represents the NES, while the right side represents the SPSA. The solid line E shows the result of the SEM under a source-based attack. The dashed lines J–M represent the curve under the contrast method.

The results of an untargeted source-based black-box attack are depicted in Figure 6A. The ensemble model exhibits weaker robustness to both NES and SPSA attacks compared to the single model, highlighting the vulnerability of the ensemble model to black-box attacks. Both the SPSA and NES approaches assume that the gradient direction of adversarial samples follows a certain probability distribution. This assumption is based on randomly sampling the gradient direction under a probability distribution, with the step size controlled by the loss value. The evaluation of the SEM under this expectation hypothesis is essentially a measure of the overlap between the gradient direction and the assumed distribution direction under the probability. In the experiment, the SEM does not demonstrate superior untargeted black-box defense effectiveness compared to the smoothed ensemble, suggesting that the SEM based on different smoothing parameters may be more susceptible to high variance noise expectations (set δ as 1 for contrast method). We believe that this characteristic can be attributed to the high ensemble probability of an unsmoothed model or a smoothed model with low variance. As a result, the defensive effectiveness of SEM is not as impressive as that of the ensemble-smoothed model in terms of probability. This result highlights the influence of the smoothing model collection on the attack performance with respect to the smoothing parameter distribution.

In comparison, the results for the targeted source-based black-box attacks that show a decrease in overall accuracy are shown in Figure 6B. Nevertheless, the same conclusion regarding robustness can be drawn. The sensitivity of the model to specific noise distributions was analyzed through experiments with black-box attacks, and it was found that the smoothing model resulted in improved defense performance against adversarial samples based on specific noise distribution assumptions. However, the model's susceptibility to noise with varying parameters under different smoothing parameters limits its defense capabilities. Such noise assumptions are independent of the true gradient information of the model and rely primarily on changes in the model output and the number of queries. Improvements in the selection of smoothing parameters for the ensemble strategy are needed to further enhance the defensive capabilities.

### 4.5. Robustness analysis based on the stochastic ensemble strategy

This section examines the effect of ensemble quantity and heterogeneity on the robustness of the proposed method. Specifically, we compare ensembles with quantities of 1, 2, and 3 to those with quantities of 6, 7, and 8 (multi_ensemble). In addition, we compare a stochastic ensemble consisting of a single-architecture CNN with different smoothing parameters. To ensure comparable prediction accuracies with our method, we choose the WRN (Zagoruyko and and Komodakis, 2016b) as the single-architecture neural network (single_architecture). To expand the stochastic ensemble model collection space and introduce model gradient variations, we smooth the WRN using seven different smoothing parameters (0.12, 0.15, 0.25, 0.5, 0.75, 1.0, and 1.25) under Gaussian noise *via* stability training (Li B. et al., 2019), semi-supervised learning (Carmon et al., 2019), and pre-training (Hendrycks et al., 2019). The resulting stochastic ensemble, consisting of a single-architecture CNN, shows heterogeneity in its smoothing attributes.

**Figure 7:**
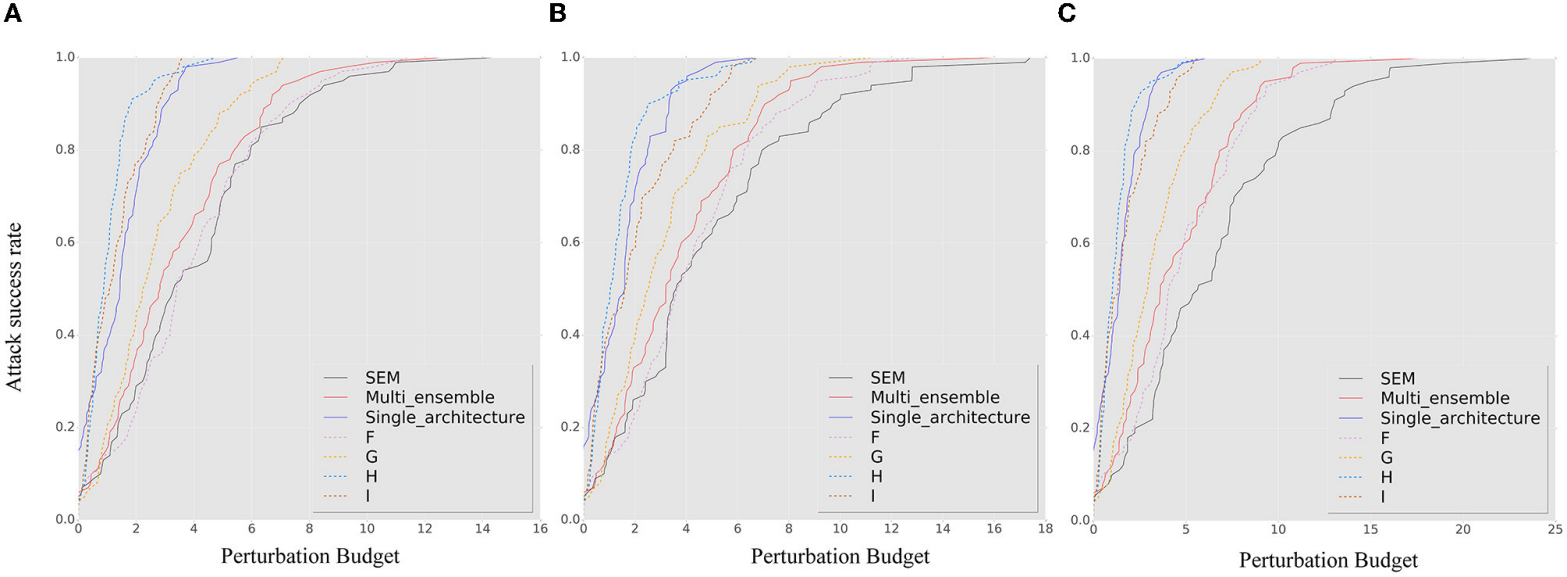
The *ASR-vs.-distortion* curves for untargeted white-box attacks under different attack methods and ensemble strategies: **(A)** BIM, **(B)** MIM, and **(C)** PGD. The solid lines represent the *ASR-vs.-distortion* curves under different ensemble strategies, while the dashed lines represent the same curves under the contrast method for comparison.

**Figure 8:**
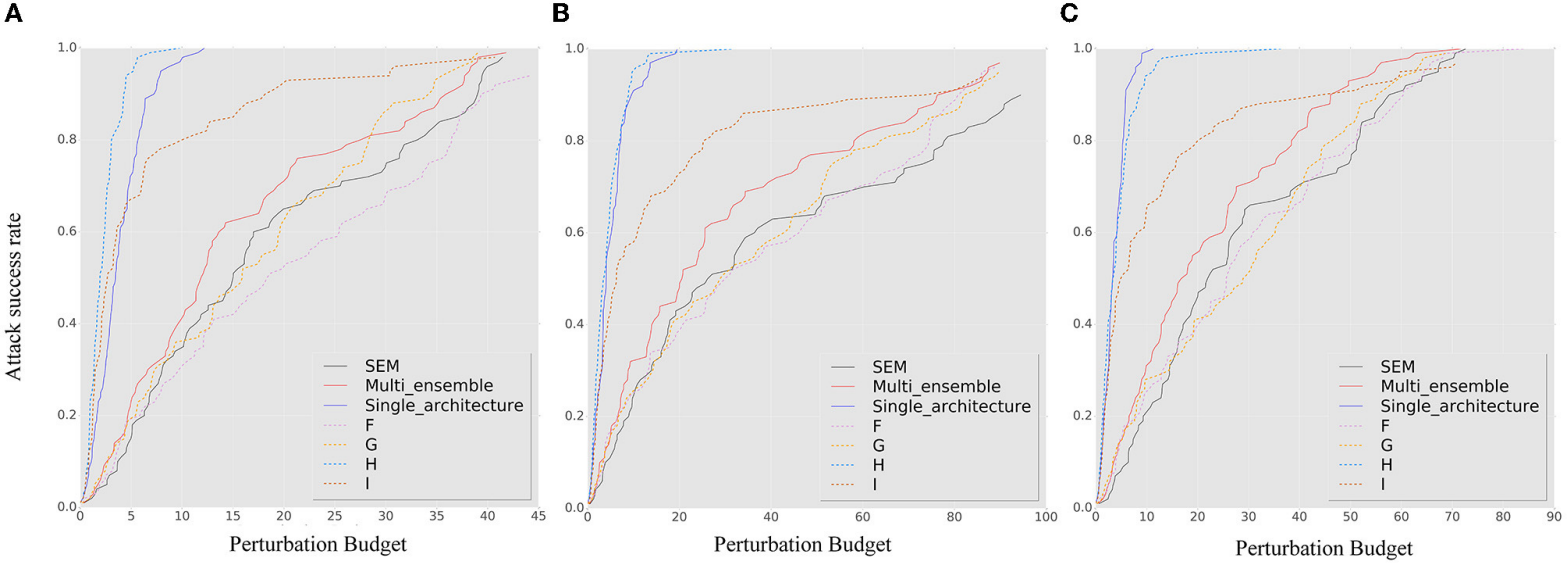
The *ASR-vs.-distortion* curves for white-box targeted attacks under different attack methods and ensemble strategies: **(A)** BIM, **(B)** MIM, and **(C)** PGD. The solid lines represent the *ASR-vs.-distortion* curves under different ensemble strategies, while the dashed lines represent the same curve under the contrast method.

Figures 7, 8 show the results of our robustness evaluation using different ensemble strategies. The negative impact of ensemble quantity on robustness is evident, as shown by the red solid line. As explained in Section 3.3, a larger ensemble quantity leads to reduced gradient differences and increased transferability of adversarial samples across ensemble iterations. The blue solid line in Figure 7 indicates that architectural heterogeneity has a greater impact on the adversarial robustness of the SEM. When there are no architectural differences between the ensemble models, even in the random smoothing case, the SEM can actually increase vulnerability to adversarial samples.

Figure 8 confirms that an SEM without architectural heterogeneity is even more vulnerable than an ensemble model. Viewing the ensemble strategy of SEM as a form of dropout operation (Baldi and Sadowski, 2013), we observe that when the ensemble quantity is large and there is insufficient architectural diversity, the SEM method becomes a regularization technique that conversely enhances the capability of adversarial samples, especially under targeted attack.

## 5. Conclusion

This study proposes a dynamic defense method for the generalized robustness of deep neural networks based on random smoothing. This dynamic nature based on the ensemble system is a change from the perspective of the existing random method from the model level to the system level. The ensemble attributes are considered as the changeable factor and dynamically adjusted during the inference prediction phase. The proposed method has the characteristics of diversity, randomness, and dynamics to achieve the probabilistic attribute dynamic defense for adversarial robustness without damaging the accuracy of clean samples. Through an optimal search of perturbation values under different attack capabilities, attack methods, and attack targets according to the degree of the real-time ability of an attacker to obtain knowledge of the model collection and gradients, a comprehensive evaluation under CIFAR10 preliminarily demonstrates that when the image distortion is small, even the attacker with the highest attack capability cannot easily exceed the attack success rate associated with the ensemble smoothed model, especially under untargeted attacks.

The robustness of our proposed method relies heavily on the heterogeneity and confidentiality of the model collection. Through experimental setups under different attack scenarios, this study also finds that the proposed SEM can achieve better robustness by limiting the ability of the adversary. Therefore, based on these findings, future studies will be conducted (1) to further improve the robustness against white-box attacks, adaptive control of the ensemble changes based on attack detection is a crucial research direction; (2) under the query-based black-box analysis, the smooth parameter selection probability of the ensemble strategy is a crucial optimization direction for this study; (3) for practical applications, both the number of parameters of the model and the forward efficiency of the ensemble prediction should be considered. In this study, the robustness is evaluated on the CIFAR10 dataset, but there are practical application problems because of the large training cost. Therefore, the light weight of the ensemble model is an important research direction.

## Data availability statement

The original contributions presented in the study are included in the article/supplementary material, further inquiries can be directed to the corresponding author.

## Author contributions

RQ: conceptualization, validation, software, and writing—original draft. LW: methodology, resources, and supervision. XD: funding acquisition, investigation, and supervision. PX: supervision. XC: project administration and funding acquisition. BY: writing—reviewing and editing. All authors contributed to the article and approved the submitted version.
